# Anisotropic Optical Response of Silver Nanorod Arrays: Surface Enhanced Raman Scattering Polarization and Angular Dependences Confronted with Ellipsometric Parameters

**DOI:** 10.1038/s41598-017-04565-0

**Published:** 2017-06-27

**Authors:** Martin Šubr, Martin Petr, Ondřej Kylián, Josef Štěpánek, Martin Veis, Marek Procházka

**Affiliations:** 1 Charles University, Faculty of Mathematics and Physics, Institute of Physics, Ke Karlovu 5, 121 16 Prague, Czech Republic; 2 Charles University, Faculty of Mathematics and Physics, Department of Macromolecular Physics, V Holešovičkách 2, 180 00 Prague, Czech Republic

## Abstract

Silver nanorod arrays prepared by oblique angle deposition (AgOADs) represent versatile, simple and inexpensive substrates for high sensitivity surface enhanced Raman scattering (SERS) applications. Their anisotropic nature suggests that their optical responses such as the SERS signal, the depolarization ratio, reflectivity and ellipsometric parameters critically depend on the states of polarization, nanorod angular arrangement and specific illumination-observation geometry. SERS polarization and angular dependences of AgOADs were measured using methylene blue (MB) molecule. Our study constitutes, to our knowledge, the most detailed investigation of such characteristics of plasmonic nanostructures to date. This is due to the 90°-scattering geometry used in which two out of three Euler angles determining the nanorod spatial orientation and four polarization combinations can be varied simultaneously. We attributed the anisotropic optical response to anisotropic (pseudo)refractive index caused by different periodicity of our structures in different directions since the plasmonic properties were found rather isotropic. For the first time we demonstrate very good correspondence between SERS intensities and ellipsometric parameters for all measured configurations as compared on the basis of the surface selection rules. Obtained results enable quantitative analysis of MB Raman tensor elements, indicating that the molecules adsorb predominantly with the symmetry axis perpendicular to the surface.

## Introduction

Plasmonic nanostructures possessing anisotropic morphology are expected to be anisotropic in terms of localized surface plasmon resonances, which implies that their optical responses such as absorbance or the enhancement factor should depend on the incident as well as the scattered polarization. In a typical Raman experiment, measurements employing combinations of different polarizations (or possibly different scattering angles) allow to compute the Raman depolarization ratio^1–3^ which gives information on the orientation-averaged components of the Raman tensor of the analyte and consequently on the symmetry of the vibration involved or on the preferential molecular orientation on smooth surfaces (“surface selection rules”)^4, 5^. However, in SERS, the relationship between the symmetry of the Raman tensor of the analyte and the SERS depolarization ratio is often completely overwhelmed by polarization-dependent coupling of the incident (or scattered) field to a given nanostructure^3, 6, 7^. This effect is probably most pronounced for dimers^8–11^, *i.e*. two closely spaced nanoparticles between which a molecule is embedded. It was found that the field component parallel to the dimer axis exceeds the perpendicular component by up to ~5 orders of magnitude^9, 10, 12^, which was explained by strong interparticle coupling between adjacent nanoparticles (however, in practice, a factor around 5–20 is more likely)^8, 13–15^. Thus, denoting α as the angle between the dimer axis and the incident polarization wavevector, the local field should scale as ~cos α, the local intensity as ~cos^2^ α and the SERS enhancement factor as ~cos^4^ α in the most common E^4^ approximation, which was verified experimentally many times. A more in-depth approach was derived in the literature^2, 3^ where a formula linking the Raman and SERS depolarization ratios beyond the E^4^ approximation was derived. However, the possibility of using SERS to probe the Raman tensor of molecules or their spatial arrangement still remains in question since it is difficult to verify the formula experimentally^2, 16, 17^.

Polarization characteristics of SERS spectra have already been investigated for a wide range of plasmonic platforms possessing anisotropic morphology, besides nanoparticle dimers Ag nanocubes^18^, arrays of silver nanoparticle rows^13^, gold nanoassemblies^19^ etc. Frequently-studied are also arrays of elongated nanoparticles such as nanorods^20–23^, nanowires^17, 24–26^, nanoantennas^27^ and nanorattles^28^. Experimental results published so far suggest that the optical response for light polarized parallel/perpendicular to the long axis of the nanoobjects (related to excitation of transverse plasmon modes (TM) or longitudinal plasmon modes (LM), respectively) is indeed different. However, the SERS intensity profile with varying angle/polarization is a function of a wide range of parameters, such as the dimensions of the metallic objects, their aspect ratios and spatial arrangement, material (Ag or Au), the exciting wavelength or orientation of the probe molecules on the surface, which resulted in the last years in some seemingly contradictory results. For example, according to Dluhy and co-workers^21^, the ratio of SERS responses in the directions parallel/perpendicular to the nanorods was found to be around 0.8, which was attributed to the lateral arrangement of the nanorod lattice and strong electromagnetic coupling between adjacent metallic nanorods instead of preferential molecular orientation of the probe molecule on the surface. Similar trends were observed for Ag and Au nanowires^17, 24–26^ where the SERS intensity ratios for light polarized perpendicular/parallel with respect to the nanorod axes was found around 5 and explained by excitation of a new plasmon mode trapped in the interstices between adjacent, parallel wires, similarly to the case of a dimer. However, this only happens if the interwire distance is sufficiently small (<10 nm)^24^. By contrast, the response of nanorods or antenna-like structures^22, 23, 27, 28^ were all found to be dominated by the longitudinal plasmon modes. Such results were, on the other hand, usually rationalized by intense local electromagnetic fields emanating from points of high curvature, such as nanorod tips (“lightning rod effect”).

Since the vast majority of experiments have been performed only in the backscattering geometry, a detailed inspection on the angular dependence of the SERS signal is still missing. However, possible optimization of plasmon-based sensors for maximum signal enhancement relies, among other things, on the right choice of the exciting and/or the scattering angle^29–33^. A modified Greenler model based on classical electrodynamic dipole radiation was used to explain the anisotropic nature of the Ag nanorods, producing maximum SERS intensities at approximately 45° relative to the surface normal as measured in a backscattering geometry^20, 34^. This model was treating the surface of the nanorod (length ~868 nm) as planar, neglecting the diffraction effect and calculating near-field intensities using the Fresnel equations. However, nanorods of subwavelength dimensions can be tricky to treat as planar as demonstrated by Benson and co-workers^35^ where a strong difference between the optical constants of the nanorod films and those of the constituent materials was found using generalized ellipsometry. Such nanorods exhibited biaxial properties with the complex refractive index different for different orientations of the incident angle with respect to the nanorods. The anisotropy is most significant predominantly at plasmonic resonances^36^.

In spite of numerous studies and approaches aiming to elucidate the optimum conditions for SERS signal enhancement and studies dealing with ellipsometry characteristics of plasmonic substrates, a detailed relationship between SERS intensities and ellipsometry parameters is still missing in the literature. In this paper, polarization and angular characteristics of methylene blue (MB) adsorbed on silver nanorod arrays prepared by oblique angle deposition (AgOADs) are investigated. These nanostructures represent uniform, reproducible, large-area SERS-active substrates with high SERS enhancement and exhibit plasmon resonance over a very broad range of wavelengths^37, 38^. We demonstrate very good correspondence between SERS intensities, interpreted in terms of the surface selection rules, and ellipsometric parameters for all polarization states and angular arrangements used. Information on the relative magnitudes of the Raman tensor elements is extracted and a possible adsorptive stance of the MB molecules on the nanorod surface is inferred.

## Methods

### Preparation of AgOADs

**Figure 1:**
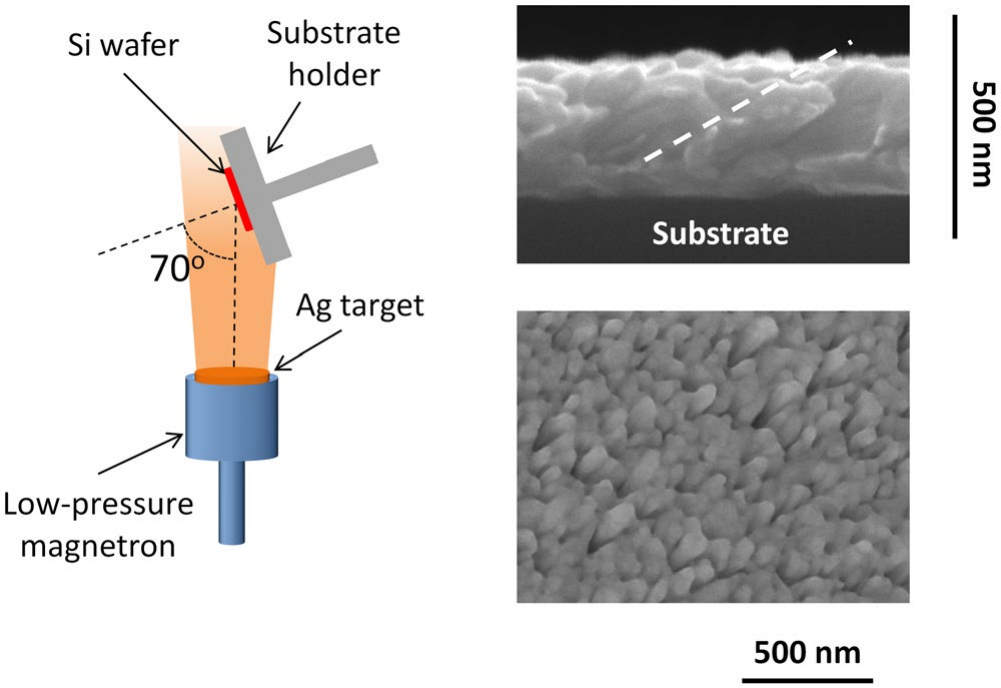
Schematic illustration of the oblique angle deposition and SEM images of a Ag nanorod array (cross-sectional and top view). In the cross-sectional view, direction of the nanorod growth is indicated by a dashed line.

Fabrication of AgOADs was performed by low-pressure magnetron sputtering of a silver target onto Si wafer support using the procedure described in more detail in our previous work^39^. The deposition angle was 70° with respect to the surface normal, corresponding to nanocolumns tilted at an angle *β* around 55° as predicted by the Tait’s rule^40^. The deposition time was 15 minutes, which led to the mean diameter of individual Ag nanorods of 60 nm, the mean distance between their centers 150 nm and the height of the nanorod array around 300 nm as can be seen in Fig. 1. There, a cross-sectional and top view of AgOADs acquired by scanning electron microscopy (SEM, TESCAN Mira 3, 15 kV accelerating voltage) are presented. All substrates were kept in a vacuum chamber overnight to prevent their contamination from ambient environment.

### SERS Measurements

**Figure 2:**
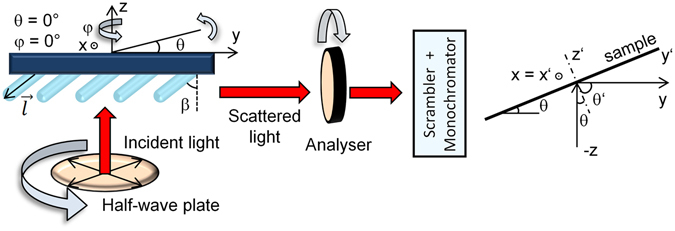
Scheme of the experimental geometry.

SERS spectra were acquired using a custom-built Raman spectroscopic system operating in the 90°-scattering geometry according to the scheme in Fig. 2. This system was equipped with a frequency-doubled Nd:YVO_2_ laser providing the excitation wavelength 532 nm, an 1800 grooves/mm grating and a liquid-nitrogen-cooled CCD detector (Princeton Instruments). The laser beam was partly focused using an anti-reflective coated lens (BK7 glass, focal length 400 mm) to a spot of ~10 mm^2^ at an angle *θ* = 45°. The SERS signal was collected using the Pentacon objective 1.8/50. Since the 532 nm excitation wavelength approaches the slope of the electronic absorption band of MB, obtained SERS spectra should be considered as pre-resonance ones. A holographic notch-plus filter (Kaiser) was placed in front of the entrance slit of the monochromator to remove the Rayleigh line from the scattered light. Light polarization falling on the sample was altered by a half-wave plate (Thorlabs) and an analyser was placed between the sample and the monochromator to allow only the light polarized in one plane fall on the detector. In order to overcome different grating responses for different light polarizations, a scrambler was inserted between the analyser and the monochromator. Correct function of the scrambler and other optical components was checked using CCl_4_ bands (measured in a cuvette) with a well-known polarization-dependent behaviour^1^.

For better description of the experiments, we adopted the laboratory-fixed coordinate system with axes lined up as sketched in Fig. 2. We call the direction along the wavevector of the incident beam (“vertical”) the *z* direction, analogously the *y* direction along the wavevector of the scattered beam and the *x* direction perpendicular to *y* and *z* (perpendicular to the plane of sheet). In this notation, *yz* determines the scattering plane. SERS spectra of MB were retrieved with varying tilting angle of the substrate *θ* (corresponding to rotation about the *x* axis) and the azimuthal angle φ (corresponding to rotation about the *z* axis). Four angles *φ* were used in our measurements (0°, 90°, 180° and 270°) with the angle *θ* bound between 20° and 70° (increment 4°; with exclusion of the interval between ~40°–50° in order to avoid direct reflection from the surface falling on the detector). The unit vector $\overrightarrow{l}$ lying in the direction of growing nanorods takes under 4 respective orientations of the angle *φ* in the laboratory-fixed coordinate system the following form:

1 $$\phi =0^\circ :\,\overrightarrow{{l}_{1}}=(0,\,\sin (\theta -\beta ),-\cos (\theta -\beta )),$$

2 $$\phi =90^\circ :\overrightarrow{\,{l}_{2}}=(\sin \,\beta ,\,\sin \,\theta \,\cos \,\beta ,-\cos \,\theta \,\cos \,\beta ),$$

3 $$\phi ={180}^{\circ }:\overrightarrow{{l}_{3}\,}=(0,\,\sin (\theta +\beta ),-\cos (\theta +\beta )),$$

4 $$\phi =270^\circ :\overrightarrow{\,{l}_{4}}=(-\sin \,\beta ,\,\sin \,\theta \,\cos \,\beta ,-\cos \,\theta \,\cos \,\beta ).$$

To better describe changes in SERS intensities with both angles, we further introduce the primed (sample-fixed) Cartesian coordinate system *x*′, *y*′, *z*′ where *x* = *x*′ and *z*′ specifies at any instant the substrate normal. Since there are two basic possibilities of setting the polarization of the incident beam as well as the scattered beam, a total of 4 different polarization combinations arise, which we will abbreviate as:

$$I({e}_{x}^{exc},{e}_{x}^{det})={I}_{vv},\,\,\,\,\,I({e}_{y}^{exc},{e}_{x}^{det})={I}_{hv},\,\,\,\,\,I({e}_{x}^{exc},{e}_{z}^{det})={I}_{vh},\,\,\,\,\,I({e}_{y}^{exc},{e}_{z}^{det})={I}_{hh}$$

with the first subscript standing for the exciting light and the second subscript standing for the scattered (detected) light; *v* stands for vertical (with respect to the scattering plane) and *h* for horizontal polarization. One of the four above-mentioned polarization arrangements together with angles *θ* and *φ* unambiguously define the experimental configuration for SERS response measurements.

Before SERS measurements, the AgOADs were cut into ~1 cm × 1 cm pieces, immersed in 1 × 10^−6^ M MB solution (Sigma-Aldrich) for 1 hour, then removed and dried with an air stream. The samples were glued to a supporting glass slide and fixed to a substrate holder capable of rotation about the *x* axis (see Fig. 2). The exact *y* position of the holder was set so that the incident beam would intersect the *x* axis to achieve optimal focus. Moreover, its *z* position was slightly readjusted after each change in the angle *θ* if needed to retain the laser spot at any instant exactly in the objective axis and to obtain the highest SERS signal. To keep the sample heating and photodecomposition at a bare minimum, the laser power was set to 100 mW, which resulted in the power density around 1 W/cm^2^. To increase precision of our measurements, measurement in each of the 4 polarization arrangements was repeated twice (4 spectra *I*_*vv*_, *I*_*hv*_, *I*_*vh*_, *I*_*hh*_ were measured in a given order and immediately after in the exactly opposite order) and the geometric mean of two corresponding spectra was used for further analysis to account for slight signal diminishment with time. All spectra were recorded using 30 s acquisition time (1 s exposition × 30 accumulations) and calibrated against a neon lamp.

### Spectral Ellipsometry Measurements

AgOADs were characterized using a commercially available spectroscopic ellipsometer Woollam RC2. This ellipsometer employs dual rotating compensator enabling standard ellipsometry, generalized ellipsometry, Mueller-matrix ellipsometry and reflectivity measurements in the energy spectral range from 0.7 to 6 eV. The reflectivity spectra were calibrated with respect to the oxidized silicon wafer. Woollam ellipsometric software CompleteEase was used to analyse the experimental data.

### Theoretical Description of the SERS Response

According to Moskovits’ surface selection rules, an adsorbed molecule may be thought to be illuminated by two beams, a direct beam and the beam reflected from the surface, which superimpose coherently^4, 5^. By analogy, the total scattered radiation results from interference between a directly scattered beam and the one experiencing a reflection from the surface. Having adopted the system of coordinates *x*′, *y*′, *z*′ as shown in Fig. 2, the surface selection rules for the 90°-scattering geometry read (see Supplementary Information for more details):

5 $${\rho }_{1}=\frac{{I}_{hv}}{{I}_{vv}}=\frac{{|{\alpha }_{xy}^{^{\prime} }(1+{r}_{s}^{^{\prime} })(1-{r}_{p})\cos \theta +{\alpha }_{xz}^{^{\prime} }(1+{r}_{s}^{^{\prime} })(1+{r}_{p})\sin \theta |}^{2}}{{|{\alpha }_{xx}^{^{\prime} }(1+{r}_{s})(1+{r}_{s}^{^{\prime} })|}^{2}},$$

6 $${\rho }_{2}=\frac{{I}_{vh}}{{I}_{vv}}=\frac{{|{\alpha }_{yx}^{^{\prime} }(1+{r}_{s})({r}_{p}^{^{\prime} }-1)\cos \theta ^{\prime} +{\alpha }_{zx}^{^{\prime} }(1+{r}_{s})(1+{r}_{p}^{^{\prime} })\sin \theta ^{\prime} |}^{2}}{{|{\alpha }_{xx}^{^{\prime} }(1+{r}_{s})(1+{r}_{s}^{^{\prime} })|}^{2}},$$

7 $$\begin{array}{c}{\rho }_{3}=\frac{{I}_{hh}}{{I}_{vv}}=\frac{|{\alpha }_{yy}^{^{\prime} }(1-{r}_{p})({r}_{p}^{^{\prime} }-1)\cos \,\theta \,\cos \,\theta ^{\prime} +{\alpha }_{yz}^{^{\prime} }(1+{r}_{p})({r}_{p}^{^{\prime} }-1)\sin \,\theta \,\cos \,\theta ^{\prime} }{{|{\alpha }_{xx}^{^{\prime} }(1+{r}_{s})(1+{r}_{s}^{^{\prime} })|}^{2}}\\ \quad \quad \quad \quad \quad +\,\frac{{\alpha }_{zy}^{^{\prime} }(1-{r}_{p})(1+{r}_{p}^{^{\prime} })\sin \,\theta ^{\prime} \,\cos \,\theta +{\alpha }_{zz}^{^{\prime} }(1+{r}_{p})(1+{r}_{p}^{^{\prime} })\sin \,\theta {\sin \theta ^{\prime} |}^{2}}{{|{\alpha }_{xx}^{^{\prime} }(1+{r}_{s})(1+{r}_{s}^{^{\prime} })|}^{2}},\end{array}$$

where *θ* is the incident angle, identical to the inclination angle sketched in Fig. 2, *θ*′ = 90°−*θ* in our geometry, *r*_*s*_ and *r*_*p*_ are the Fresnel reflection coefficients related to the frequency of the incident radiation, their primed counterparts refer to the frequency of the scattered radiation and *α*′_*ij*_ refers to orientation-averaged components of the Raman tensor of the adsorbed molecule in the primed frame of reference. In Eqs 5–7, we introduced the depolarization ratios *ρ*_1_, *ρ*_2_ and *ρ*_3_, all of them normalised to *I*_*vv*_ since this polarization arrangement provides information only on one term of the Raman tensor. This approach is more convenient because it enables to exclude the insignificant multiplicative constant relating Raman intensities to specific combinations of squares of Raman tensor elements^1^. The Fresnel coefficients were retrieved from optical (pseudo)parameters of the sample as measured by spectral ellipsometry and used for fitting the experimentally measured depolarization ratios on the left side of Eqs 5–7 via relative ratios of respective Raman tensor elements.

## Results and Discussion

### SERS Polarization and Angular Dependences

**Figure 3:**
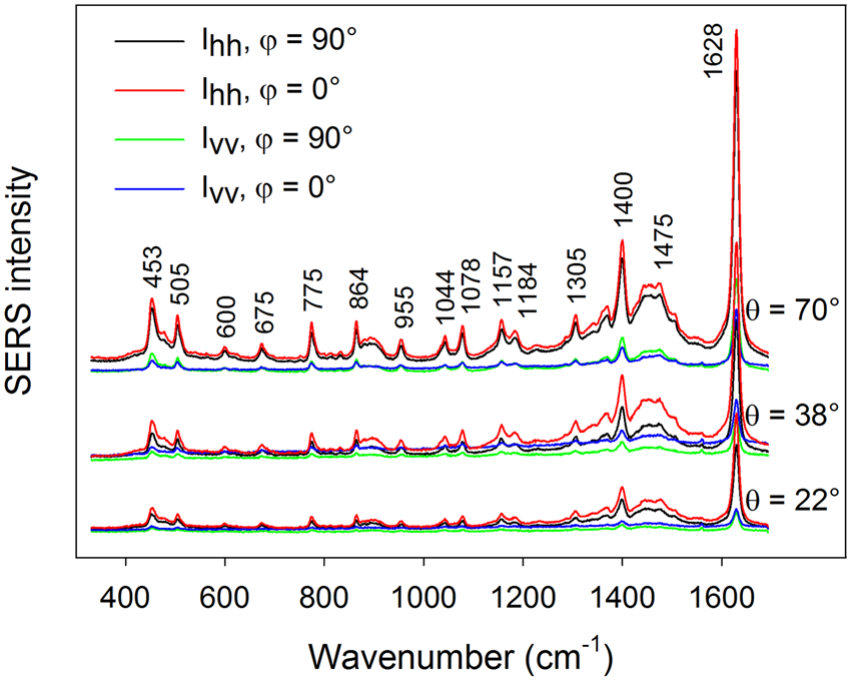
Examples of MB SERS spectra obtained under different configurations. Spectra for different angles $\theta$ are offset for clarity.

**Figure 4:**
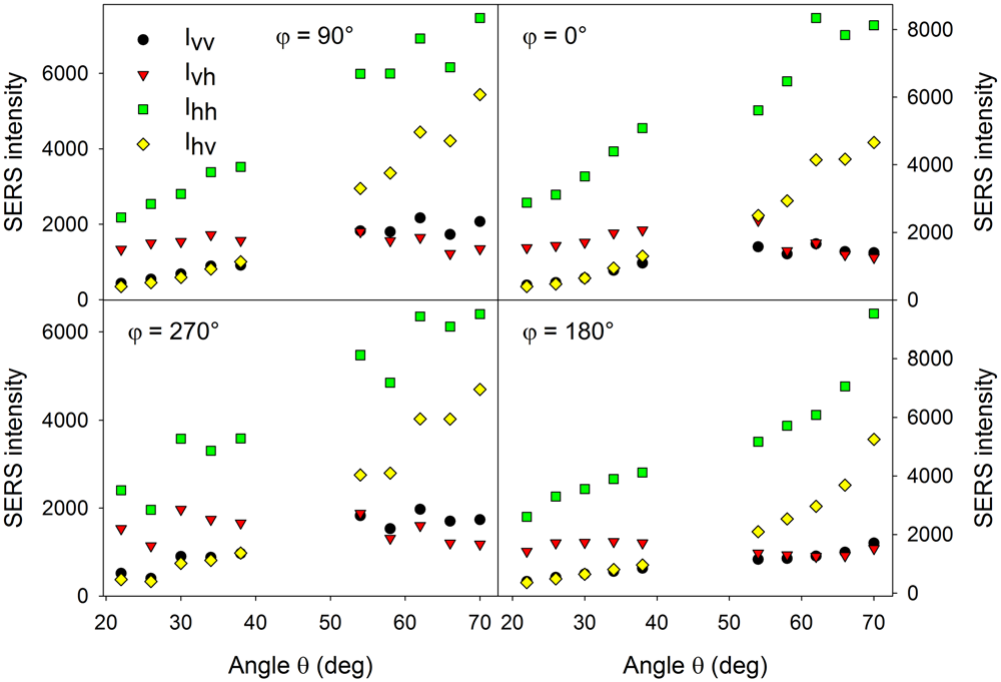
Variation in MB SERS intensities with angles $\theta$, $\phi$ and polarization arrangement for the 1628-cm^−1^ band.

Examples of MB SERS spectra obtained under different configurations are given in Fig. 3. A more detailed account of varying SERS intensities with varying angles *θ*, *φ* and polarization arrangement is given in Fig. 4. These intensities, assumed as height of the 1628-cm^−1^ peak above spectral background, vary up to ~1 order of magnitude when measured under different configurations. Since the surface area is covered with occasional hot-spot sites, we tried to estimate the uncertainty in the SERS intensities. Occurrence frequency of hot-spot sites complies well with the Poisson distribution^38^, however, the actual number of hot-spot sites affected by the laser spot is expected to average out on the mm-scale. Supposing 4 hot-spot sites over 2500 µm^2^ as determined in our previous work, it makes ~16000 hot-spot sites across the laser spot with the standard deviation of 16000^1/2^ = 126 and the relative standard deviation of the SERS intensity <1%. Uncertainty in our measurements was therefore caused mainly by slight sample photodecomposition in the course of our measurements, estimated at ~5% (i.e. ~10% uncertainty in the depolarization ratios). In order to obtain a deeper understanding of varying SERS intensities with different experimental configurations and to possibly identify subtle spectral changes such as varying relative intensities across different bands, factor analysis (FA) was employed (see Supplementary Information, Fig. S1). FA results suggest that only the first subspectrum is sufficient so that the original spectral information is retained within the noise level. Therefore, all observable MB bands in Fig. 3 exhibit the same polarization and angular-dependent behaviour. This is in agreement with the fact that most observable bands in the spectrum are of the same symmetry^41^. For the sake of comparison, absorption spectra of MB, polarization-resolved Raman spectra measured in a water solution under non-SERS conditions and corresponding depolarization ratios were also retrieved (see Supplementary Information, Fig. S2).

**Figure 5:**
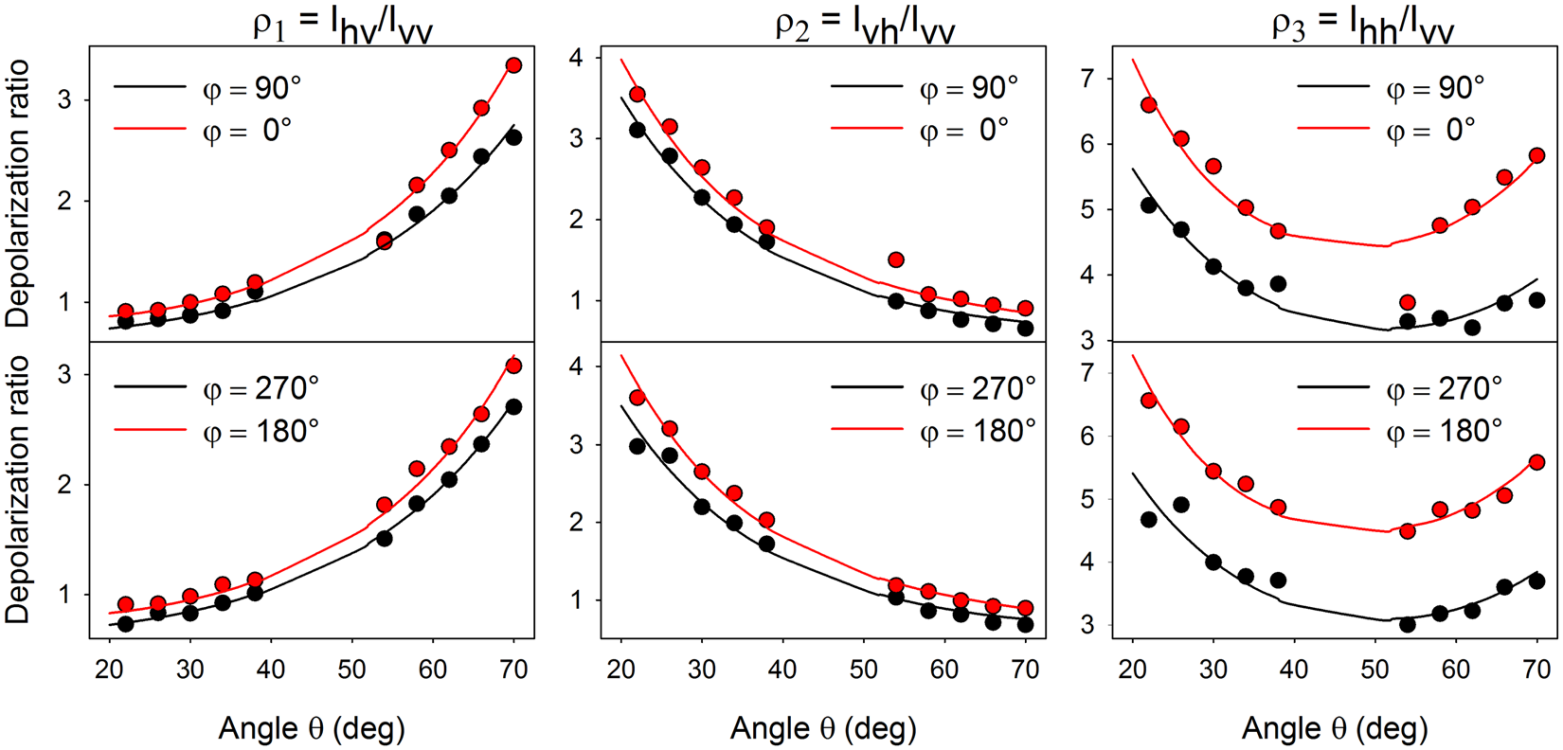
Depolarization ratios of the 1628-cm^−1^ MB band for different angular arrangements (colour points) and their fit by the surface selection rules with pseudo-refractive indices obtained from ellipsometry measurements (lines).

Obtained polarization and angular characteristics may be attributed to many different factors, such as: (i) different surface plasmon coupling efficiency between the incident/scattered laser field and silver nanorods with changing polarization/wavevector direction (“plasmonic anisotropy”), (ii) interference between the incident/scattered and reflected radiation as dictated by the surface selection rules, (iii) effectiveness of collection of the scattered radiation and different laser spot size with a given angle *θ* (scaling approximately as ~cos*θ*)^42^. In order to better understand intensity changes with varying angle *θ*, Raman spectrum of a Si wafer was measured as a reference (see Supplementary Information, Fig. S3). Comparison between Raman intensities as measured using a Si wafer and corresponding theoretical values predicted by the surface selection rules revealed that the point (iii) plays a crucial role in the observable characteristics. Therefore, in order to diminish the effect of the geometrical layout, to highlight the difference between different angles *φ* and since we are interested rather in the relative ratios of MB Raman tensor elements instead of their magnitudes, the depolarization ratios *ρ*_1_, *ρ*_2_ and *ρ*_3_ were further analysed instead of intensities. Obtained results for these 3 depolarization ratios with varying angles *θ* and *φ* are shown as colour points in Fig. 5.

In order to determine the effect of the “plasmonic anisotropy”, a simple model based on “competition” between the enhancement provided by longitudinal plasmon modes (excited by polarization parallel to the long axis of the nanorods) and by transverse plasmon modes (excited by polarization perpendicular to the long axis of the nanorods) was employed at the first stage. The primary reason for this competition (and consequently alteration of the incident/scattered polarization caused by the nanostructures) is the presence of hot-spots, which may be distributed both at the edges of nanowires and at the gaps among adjacent nanorods. Knowing the long axes orientation under given angular arrangement in the laboratory-fixed coordinate system (determined by Eqs 1–4), intensities obtained in respective configurations are expected to be^6, 10, 11, 17, 21^

8 $${I}_{j}\sim [{a}_{||}{(\overrightarrow{{e}_{1}}\cdot \overrightarrow{{l}_{j}})}^{2}+{a}_{\perp }(1-{(\overrightarrow{{e}_{1}}\cdot \overrightarrow{{l}_{j}})}^{2})][{a}_{||}^{^{\prime} }{(\overrightarrow{{e}_{2}}\cdot \overrightarrow{{l}_{j}})}^{2}+{a}_{\perp }^{^{\prime} }(1-{(\overrightarrow{{e}_{2}}\cdot \overrightarrow{{l}_{j}})}^{2})],$$

where $\overrightarrow{{e}_{1}}$ is the unit incident field vector, $\overrightarrow{{e}_{2}}$ is the unit scattered field vector, $j\in \{1,2,3,4\}$ and *a*_⊥_ and *a*_*||*_ are factors determining the plasmonic response to polarization parallel/perpendicular to the long axis of the nanorods for the exciting light (unprimed values) and the scattered light (primed values). The first bracket in Eq. 8 accounts for the enhancement of the incident radiation and the latter accounts for the enhancement of the scattered radiation. Since we analyse the depolarization ratios instead of intensities, the final formulas will depend only on the ratio $r={a}_{\perp }/{a}_{||}$. We tried to fit the obtained depolarization ratios using the Eq. 8, but no value of *r* consistent for all three depolarization ratios and any of the four angles *φ* and copying the shape of the experimentally measured depolarization ratios (Fig. 5) was found (see section 4 in Supplementary Information for more details). An insightful view on failure of such a fit is provided by the depolarization ratios in Fig. 5, which exhibit marked distinction after rotating the sample by 90° about the *z*′ axis while upon rotating the sample by 180° the depolarization ratios change very little. This was expected for *φ* = 90° and 270° since in both cases the angle between the axes of the nanorods and incident/scattered wavevector is the same. More surprisingly, even the ratios for *φ* = 0° and 180° are similar (but still distinct from *φ* = 90° and 270°), truly indicating that different surface plasmon coupling efficiency of light polarized rather parallel/rather perpendicular to the nanorods has very little effect on the observable characteristics. In other words, although our nanostructures are morphologically anisotropic, the plasmonic properties around the wavelength used (532 nm) are rather isotropic and therefore it can not be the main reason for the anisotropic behaviour we observed in the SERS experiments. It suggests that hot-spots, which are primarily responsible for altering the polarization of the SERS photons, are evenly distributed both at the edges of nanowires and at the gaps among adjacent nanorods. This result indicates that the majority of the difference in the depolarization ratios after rotating the sample by 90° is attributable to different refractive indices along different directions and may be explained within the framework of the surface selection rules.

### Ellipsometry Characteristics

**Figure 6:**
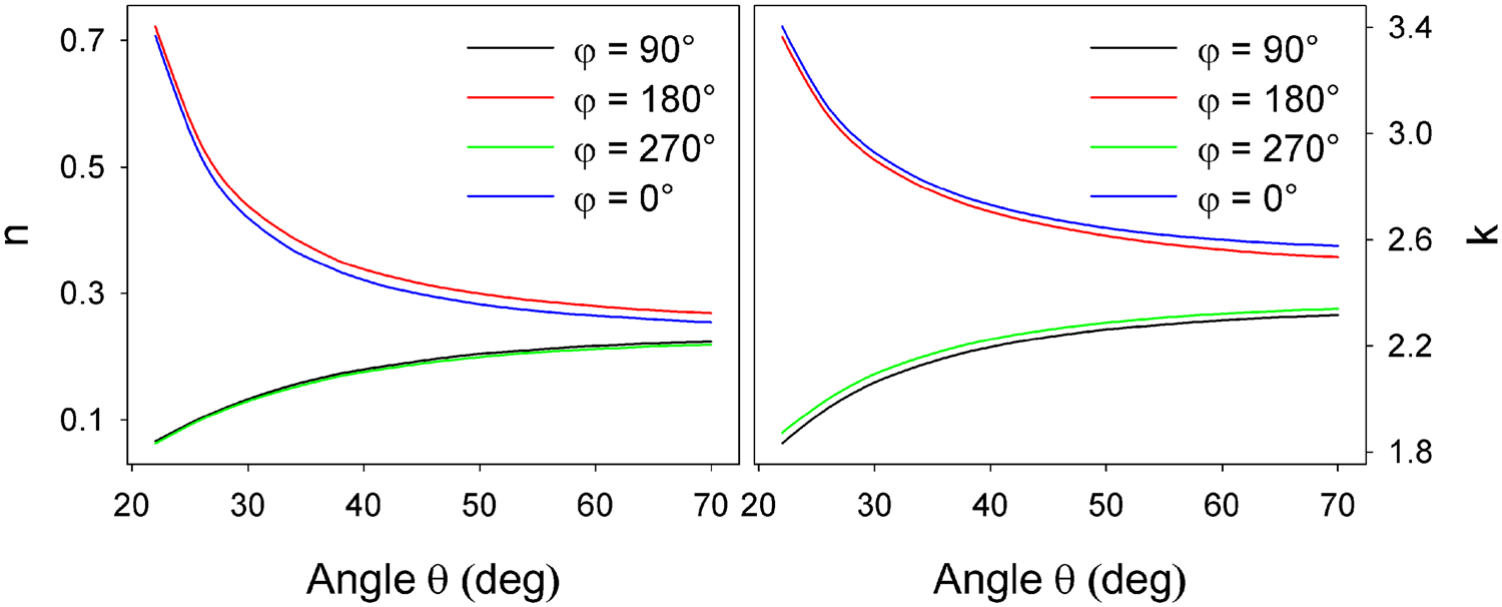
Real and imaginary part of pseudo-refractive index $\tilde{n}=n+ik$ of AgOADs and their variation with angles $\theta$ and $\phi$ as measured by standard ellipsometry. The values pertain to substrates after MB adsorption, $\lambda =532$  nm. To compare, the refractive index of the bulk silver is ${\tilde{n}}_{Ag}\sim 0.05+3.4i$ ^43^.

In order to theoretically describe the SERS response, ellipsometry measurements were performed on the nanostructures with the aim of retrieving their optical constants which enter Eqs 5–7. Ellipsometry measurements of AgOADs were carried out after MB adsorption with varying angles *θ* and *φ* in the same range as in the case of SERS measurements. From ellipsometry measurements several basic conclusions were drawn: As expected, the optical (pseudo)parameters of the nanostructured layers are strongly different from the optical constants of the constituent materials. Unlike optical constants of homogeneous materials, optical constants of silver nanorods depend (due to the presence of subwavelength structures) on the incident angle *θ* and are strongly influenced by rotating the sample by 90° about the *z*′ axis (see Fig. 6 or the section 5 in Supplementary Information for more details). On the other hand, the optical constants are virtually insensitive to rotating the sample by 180°, which is in full compliance with the SERS results. Comparison between optical (pseudo)refractive indices, obtained in standard ellipsometry measurement mode, and total reflected intensity, measured in the reflection mode (together with Mueller-matrix measurements) revealed that the structures may be treated as non-depolarizing (with only ~5% of the reflected light exhibiting depolarization, see Supplementary Information, Fig. S6) and thus analysed by the standard Jones formalism^35, 36^. Moreover, further analysis showed that the off-diagonal elements of the Jones matrix are ~10^2^ × smaller than the diagonal elements and thus may also be dismissed. This justifies application of the surface selection rules in the form given by Eqs 5–7 for theoretical analysis of the SERS response.

**Figure 7:**
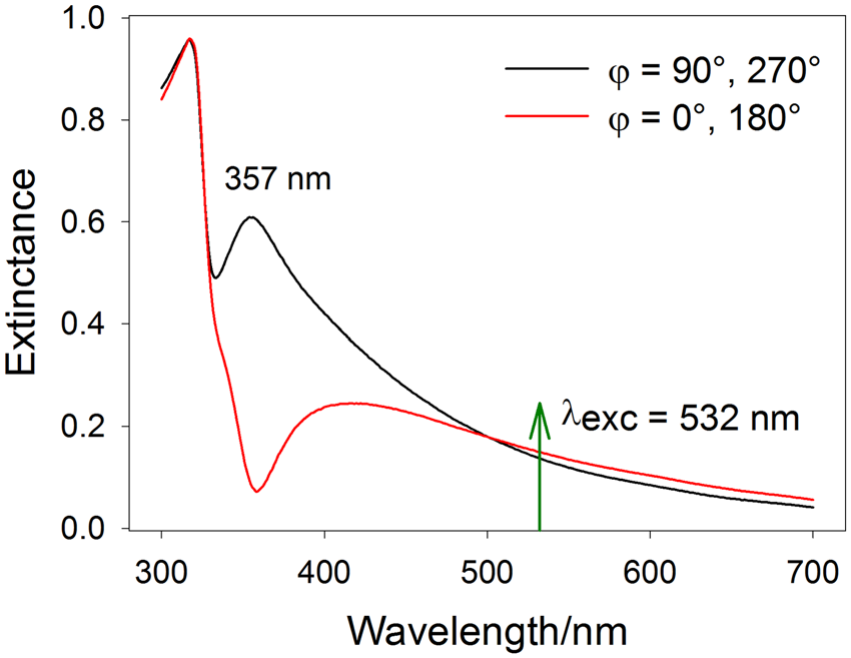
Extinctance of the AgOADs for angles $\phi =90^\circ$ (virtually identical to $\phi =270^\circ$) and $\phi =0^\circ$ (virtually identical to $\phi =180^\circ$). Extinctance ($E$) was calculated as $E=1-R$, where the reflectivity $R$ was obtained based on the values $\tilde{n}=n+ik$ from Fig. 6. The exciting wavelength is marked with a green arrow, the angle $\theta$ was 45°.

Another purpose of ellipsometry measurements was that it can reveal more detailed information on the plasmonic properties of the AgOADs. Calculated extinction spectra of our structures for given angles $\phi$ are depicted in Fig. 7. The extinctance presented there ($E$) was computed as $E=1\,-\,R$, where the reflectivity $R$ was obtained based on the values contained in Fig. 6. Extinctance features a sharp transverse plasmon peak around 357 nm and a broad band above ~400 nm, attributed to the longitudinal plasmon mode^21, 44^. The latter is less intense than expected, probably due to very high reflectivity of the sample above ~400 nm, which may be partly attributed to the supporting Si wafer. We suppose that the distinction between these two profiles may be attributed to different periodicity of our structures in the *x*′/*y*′ direction. As can be seen in Fig. 1, the nanorods are almost perfectly aligned in the direction perpendicular to the nanorod axes (the deviation of the nanorod axes from the *x*′ axis is negligible from 90°) while the tilting angle of the nanorods $\beta$ exhibits a certain distribution around the value of ~55°. Since the $\phi =90^\circ /270^\circ$ configuration is sensitive predominantly to the former case, the corresponding plasmonic resonance is expected to be rather sharp. On the other hand, in the $\phi =0^\circ /180^\circ$ arrangement, the angle made between the polarization vector and axes of respective nanorods exhibits a certain distribution which is reflected in the inhomogeneously broadened longitudinal plasmon peak above ~400 nm. Figure 7 indeed confirms that the coupling efficiency for  ﻿λ﻿ = 532 nm is almost identical for all four angles $\phi$ and therefore the difference in the SERS responses when rotating the sample about the *z*′ axis is mostly dictated by interference between the incident/scattered and reflected radiation. “Higher plasmonic anisotropy” is more likely to arise at shorter wavelengths as can be seen in Fig. 7 where the two curves differ more obviously, although more extensive research is required to confirm this. We have to note, however, that the relationship between the near-field (Raman-enhancing) properties and the far-field properties (such as extinctance of reflectance) is not straighforward in SERS and in the case of anisotropic SERS substrates no correlation between these two phenomena was found^21, 45^.

### Determination of Molecular Orientation on the Surface

Knowing the optical (pseudo)parameters of the sample, it is possible to use Eqs 5–7 to fit the measured SERS depolarization ratios (colour points in Fig. 5). Unlike the former model based on different surface plasmon coupling efficiency for different light polarization, the model using optical (pseudo)parameters fits the experimentally measured depolarization ratios very well. Moreover, these fits provide information on the relative ratios of Raman tensor elements of the probe molecule and are represented by solid lines in Fig. 5. The obtained results are summarized in Table 1.

**Table 1:** Relative magnitudes of Raman tensor elements (normalised to *α*′_*xx*_) of the 1628-cm^−1^ MB band for different angles *φ*.

*φ*	90°	0°	270°	180°	
*α*′_*xx*_	1	1	1	1	
*α*′_*xy*_	0.81	0.84	0.80	0.85	$a$
*α*′_*xz*_	0.06	0.10	0.08	0.04	
*α*′_*yx*_	0.80	0.89	0.81	0.91	$b$
*α*′_*zx*_	0.18	0.12	0.17	0.12	
*α*′_*yy*_	0.91	0.97	0.90	0.92	$c$
*α*′_*zz*_	0.01	0.05	0.01	0.06	
*α*′_*yz*_	0.01	0.09	0.01	0.01	
*α*′_*zy*_	0.24	0.16	0.23	0.16	

The values were obtained by fitting the SERS depolarization ratios using the surface selection rules. Values revealed by the fit of *ρ*_1_ are labelled as *a*, by *ρ*_2_ as *b* and by *ρ*_3_ as *c*.

Table 1 clearly demonstrates basically comparable values of ${\alpha }_{xx}^{^{\prime} }$, ${\alpha }_{yy}^{^{\prime} }$, ${\alpha }_{xy}^{^{\prime} }$ and ${\alpha }_{yx}^{^{\prime} }$ and considerably lower values of ${\alpha }_{zz}^{^{\prime} }$, ${\alpha }_{xz}^{^{\prime} }$, ${\alpha }_{zx}^{^{\prime} }$, ${\alpha }_{yz}^{^{\prime} }$ and ${\alpha }_{zy}^{^{\prime} }$. MB belongs to the C_2_ point group symmetry with the vibrational representation ${{\rm{\Gamma }}}^{3N-6}=54\,A+54\,B$ ^41^. The strongest MB band at 1628 cm^−1^ belongs to the *A* species which transform as *x*′^2^, *y*′^2^, *z*′^2^ and *x*′*y*′ (*z*′ being the axis of symmetry of the molecule)^41^. Moreover, almost all the other observable bands in the SERS spectrum exhibit very similar intensity profile and thus very similar relative magnitudes of Raman tensor elements as already discussed using FA. This conclusion is also justified by the fact that the pseudo-refractive indices change very little within the Raman shift wavelengths. Thus, for the MB orientation on the surface with the symmetry axis along the ${z}^{^{\prime} }$ direction (the plane of the fused phenyl rings perpendicular to the substrate), the ${\alpha }_{xx}^{^{\prime} }$, ${\alpha }_{yy}^{^{\prime} }$, ${\alpha }_{xy}^{^{\prime} }$ and ${\alpha }_{yx}^{^{\prime} }$ Raman tensor elements are expected to be enhanced most while the ${\alpha }_{xz}^{^{\prime} }$, ${\alpha }_{zx}^{^{\prime} }$, ${\alpha }_{yz}^{^{\prime} }$ and ${\alpha }_{zy}^{^{\prime} }$ elements should tend to zero. Therefore, we conclude that the orientation of the MB molecule on the surface is predominantly edge-on and possibly a small proportion of the molecules may take also different orientations (since the ${\alpha }_{xz}^{^{\prime} }$, ${\alpha }_{zx}^{^{\prime} }$ ${\alpha }_{yz}^{^{\prime} }$ and ${\alpha }_{zy}^{^{\prime} }$ elements are not exactly zero but still much lower than ${\alpha }_{xy}^{^{\prime} }$ and ${\alpha }_{yx}^{^{\prime} }$). This is in agreement with the literature since at higher concentrations the MB is supposed to take rather edge-on adsorptive stance on the surface with the face-on orientation preferable at lower concentrations^41, 46^. The concentration used in our experiments (10^−6^ M) is expected to be slightly above the complete surface coverage^41, 47^. We have to note, though, that the group theory (together with experimentally measured depolarization ratios) provides information only on the orientation of the molecular symmetry axis with respect to the surface, but says nothing about the distribution of the planes of the fused phenyl rings of the molecules with respect to $x^{\prime}$ or $y^{\prime}$. Thus, the non-diagonal elements of the MB Raman tensor should be viewed as orientation-averaged for an ensemble of MB molecules possessing predominantly edge-on adsorptive stance on the surface.

The similarity between the MB Raman tensor elements for four angles $\phi$ is rather peculiar since it indicates that the molecules do not follow the curved orientation of the nanostructured surface. It suggests that the molecules actually do not cover the lateral surface of the nanowires and probably aggregate in the vicinity of the nanorod tips. This may be caused by rather densely-packed nanocolumns, low porosity of the AgOADs and surface tension of the solution^31^, but obviously more in-depth experiments are required to confirm this. We propose that this could be further validated by testing different solvents, different analytes and their concentration-dependence or by preparing different nanocolumn height, porosity or constituent materials. Some of these aspects will be addressed in our further research.

Results of our fits also reveal that ${\alpha }_{xy}^{^{\prime} }\,\sim \,{\alpha }_{yx}^{^{\prime} }$, ${\alpha }_{xx}^{^{\prime} }\,\sim {\alpha }_{yy}^{^{\prime} }$ and the remaining components are very small for all angles $\phi$. This follows directly from tensor rotational transformation formulas and confirms the success of our fitting procedures since different tensor components were obtained from different fits. For example, when rotating the system by 90° about the $z^{\prime}$ axis, the ${\alpha }_{xx}^{^{\prime} }\,\,$component becomes ${\alpha }_{yy}^{^{\prime} }$, the ${\alpha }_{xy}^{^{\prime} }$ component becomes ${\alpha }_{yx}^{^{\prime} }$, the ${\alpha }_{xz}^{^{\prime} }\,\,$component becomes ${\alpha }_{yz}^{^{\prime} }$ and the ${\alpha }_{zx}^{^{\prime} }\,\,$component becomes ${\alpha }_{zy}^{^{\prime} }$ and vice versa (for details see section 7 in Supplementary Information). Table 1 demonstrates the agreement between MB Raman tensor elements even across different angles $\phi$, although obviously not all elements are obtained with the same accuracy which causes slight discrepancies. Unfortunately, that tensor components containing $z^{\prime}$ in at least one of its indices suffer from higher uncertainty, especially those obtained from the fit of ${\rho }_{3}$, which presents rather an ill-posed problem since it aims to fit four values of the Raman tensor simultaneously. In order to obtain a better criterion for reliability of our fits, we further computed the usual Raman tensor invariants (see the section 7 in Supplementary Information for more details), the corresponding depolarization ratio of MB adsorbed on AgOADs and compared these values with the values retrieved from MB Raman spectra measured in a solution. Raman tensor elements in Table 1 give the usual Raman tensor invariants for each of the angles $\phi$ as follows: ${a}^{2}=0.43\pm 0.02$, ${\gamma }^{2}=3.1\pm 0.1$ and ${\delta }^{2}=0.03\pm 0.01$. The corresponding depolarization ratio is $\rho =0.29\pm 0.01$. To compare, the depolarization ratio of MB as measured in a water solution (see the section 2 in Supplementary Information for more details) is ${\rho }_{M}=0.21\pm 0.01$ for all symmetric vibrations involved. We suppose that the difference may be attributable either to the presence of hot-spot sites or to formation of dimers/trimers at the surface which could feature a different depolarization ratio with respect to the monomers that are analysed in liquid (or possibly to a combination of the two factors). Hot-spot sites are sparsely distributed over the surface as demonstrated by spectral mapping of the surface in our previous work^38^. These sites may occur either at the edges of nanowires or at the gap among nanoparticles (with completely different polarization characteristics), but since no significant difference between efficiency of excitation of longitudinal/transverse plasmon modes was observed, we hypothesize that the hot-spot occurrences in these two locations are approximately equal. On the other hand, for a collection of randomly oriented hot-spots such as colloids, the measured depolarization ratio of all modes under SERS conditions will be ${\rho }_{SERS}=1/3$, virtually independent of the symmetry of a given Raman mode^7, 17^. When measuring in a macro-mode (with the laser spot in the order of several mm^2^), a large number of hot-spots is affected, which may still cover rather negligible proportion of the illuminated area but have a non-negligible share of the total intensity, difficult to estimate precisely. Thus, for SERS-active systems where only a certain proportion of the signal (say 50%) comes from randomly-oriented hot-spots, one can expect that the depolarization ratio of the vibrations (for which the depolarization ratio under non-SERS conditions is ~0.21) will be shifted towards 1/3. Supposedly the depolarization ratio as computed using values in Table 1 is bound between these two extremes (${\rho }_{M} < \rho  < {\rho }_{SERS})$.

In summary, a detailed analysis of the SERS polarization and angular dependences was performed on MB adsorbed on silver nanorod arrays prepared by oblique angle deposition (AgOADs). The main advantage of our approach is employment of the 90°-scattering geometry in which two out of three Euler angles determining the nanorod spatial orientation and four polarization combinations can be varied simultaneously. This enabled us to carry out the most in-depth investigation of polarization- and angular-resolved characteristics of AgOADs to date, as far as we know. Ellipsometry measurements were carried out on the nanostructures to characterize their plasmonic properties and to retrieve their optical constants which enter the surface selection rules. A detailed relationship between the SERS intensities and corresponding ellipsometric parameters was elucidated, which was still missing in the literature. Both the SERS intensities as well as ellipsometric parameters were found to exhibit strong dependence on rotating the sample by 90°, although being fairly insensitive to flipping the sample by 180°. We suppose that this distinction may be attributed to slightly different periodicity of our structures in different directions instead of different surface plasmon coupling efficiency for light polarized parallel/perpendicular to the nanorod arrays as reported in numerous studies where a comparable approach was employed for other SERS-active systems. The theoretical description of the SERS response on the basis of the surface selection rules was therefore well applicable. Fit of the obtained depolarization ratios against the optical (pseudo)parameters of the sample obtained by means of spectral ellipsometry provided relative magnitudes of MB Raman tensor elements. These results enable to gain insight into the adsorptive stance of the molecule on the surface, indicating that the MB adsorbs predominantly with the symmetry axis perpendicular to the surface. Our experimental findings contribute to better theoretical understanding of the SERS enhancement mechanism and may be useful for optimization of plasmon-based sensors for maximum signal enhancement.

## Electronic supplementary material

Supplementary Information
