# Supplementary material for: Anisotropic Optical Response of Silver Nanorod Arrays: Surface Enhanced Raman Scattering Polarization and Angular Dependences Confronted with Ellipsometric Parameters
Source: Sci Rep. 2017 Jun 27;7:4293. doi: 10.1038/s41598-017-04565-0 (PMC5487367; doi:10.1038/s41598-017-04565-0)
Supplement: Supplementary file 1 — Supplementary Information [file 41598_2017_4565_MOESM1_ESM.pdf]

Supplementary Information

Anisotropic Optical Response of Silver Nanorod Arrays: Surface Enhanced Raman Scattering Polarization and Angular Dependences Confronted with Ellipsometric Parameters

Martin Šubr,[*†] Martin Petr,[§] Ondřej Kylián,[§] Josef Štěpánek,[†] Martin Veis,[†] and Marek Procházka[*†]

† Charles University, Faculty of Mathematics and Physics, Institute of Physics, Ke Karlovu 5, 121 16 Prague, Czech Republic

§ Charles University, Faculty of Mathematics and Physics, Department of Macromolecular Physics, V Holešovičkách 2, 180 00 Prague, Czech Republic

Corresponding authors:

* M. Šubr: e-mail subr.Martin@seznam.cz

* M. Procházka: e-mail prochaz@karlov.mff.cuni.cz

1. Factor Analysis

In order to obtain a deeper understanding of varying SERS intensities with different experimental configurations and to possibly identify subtle spectral changes such as varying relative intensities across different bands, factor analysis (FA) was employed. In the singular value decomposition (SVD) algorithm, a set or N background-corrected MB SERS spectra $Y_i(v)$ ($i = 1$ corresponding to $\vartheta = 22°$,… $i = N$ corresponding to $\vartheta = 70°$; angle φ playing the role of a parameter) are projected into orthonormal set of functions $S_i(\nu)$ (referred to as subspectra) as

$$Y_i(\nu) = \sum_j W_j V_{ij} S_j(\nu), \qquad \text{(S1)}$$

where W_j $(j = 1,2, \dots N)$ is the jth singular value, representing relative statistical weight of the jth subspectrum, and V_{ij} is a unitary matrix determining relative spectral contribution of $S_j(\nu)$ in $Y_i(\nu)$.[1] FA results for spectra measured for $\varphi = 0°$ are given in Fig. S1 and suggest that only the first subspectrum is sufficient so that the original spectral information is retained within the noise level (factor dimension is 1). In other words, eq S1 simplifies to

$$Y_i(\nu) = W_1 V_{i1} S_1(\nu), \qquad \text{(S2)}$$

because higher V_{ij} coefficients ($j \geq 2$) exhibit rather random behaviour and were not reproducible in our measurements. Corresponding subspectra $S_j(\nu)$, $j \geq 2$, describe only fluctuating spectral background, extremely sensitive to slight changes in baseline subtraction, and even higher subspectra only the white noise. This is in agreement with the fact that all observable bands in the spectrum are of the same symmetry.[2]

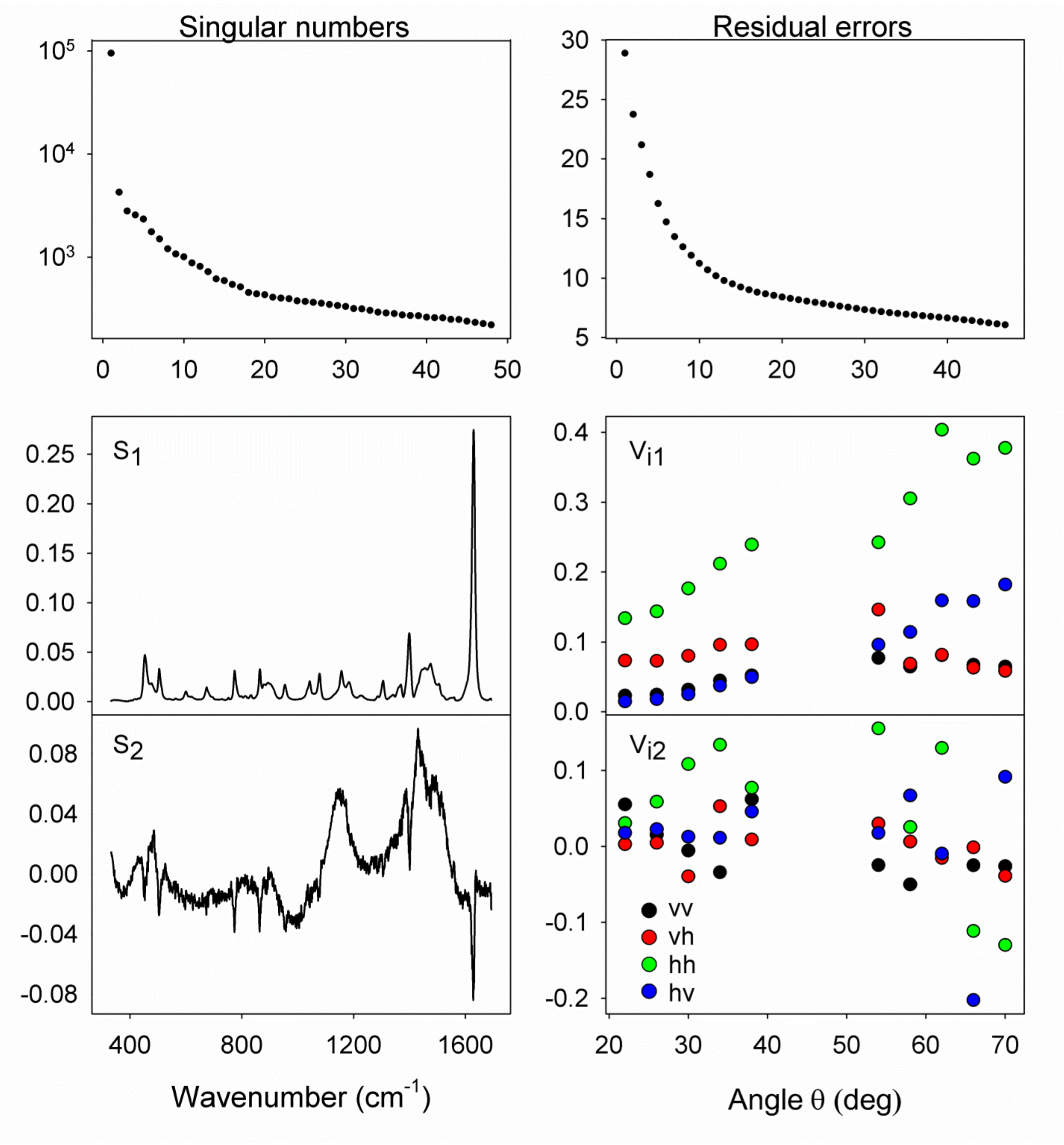

Figure S1. FA results of baseline-corrected MB SERS spectra measured at an angle $\varphi = 0°$. The subspectrum S_1 represents the basic spectral profile and the profile of the V_{i1} coefficients very well fits the intensity profile of the 1628-cm^{-1} MB band given in Fig. 4. Different polarization arrangements are distinguished by different colours. Shape of the subspectrum S_2 and profile of the V_{i2} coefficients were not reproducible in our measurements and were both extremely sensitive to slight changes in background subtraction.

2. Raman Spectra of Methylene Blue

For the sake of comparison, polarization-resolved Raman spectra of MB measured in a water solution under non-SERS conditions were retrieved. These spectra are depicted in Fig. S2. In the case of randomly oriented molecules and the 90°-scattering geometry, the ratio of cross-polarized (in principle, any of I_{hv}, I_{vh} or I_{hh} polarization arrangements) to parallel-polarized (I_{vv} arrangement) Raman intensities defines the molecular depolarization ratio ρ_M. The depolarization ratios of the most intense A-type bands were computed to be $\rho_M = 0.21 \pm 0.01$.

Absorption spectra of MB in the same concentration were also retrieved. MB absorption spectra feature a maximum at 665 nm and a shoulder around 618 nm, both of them being characteristic of a monomeric form[3].

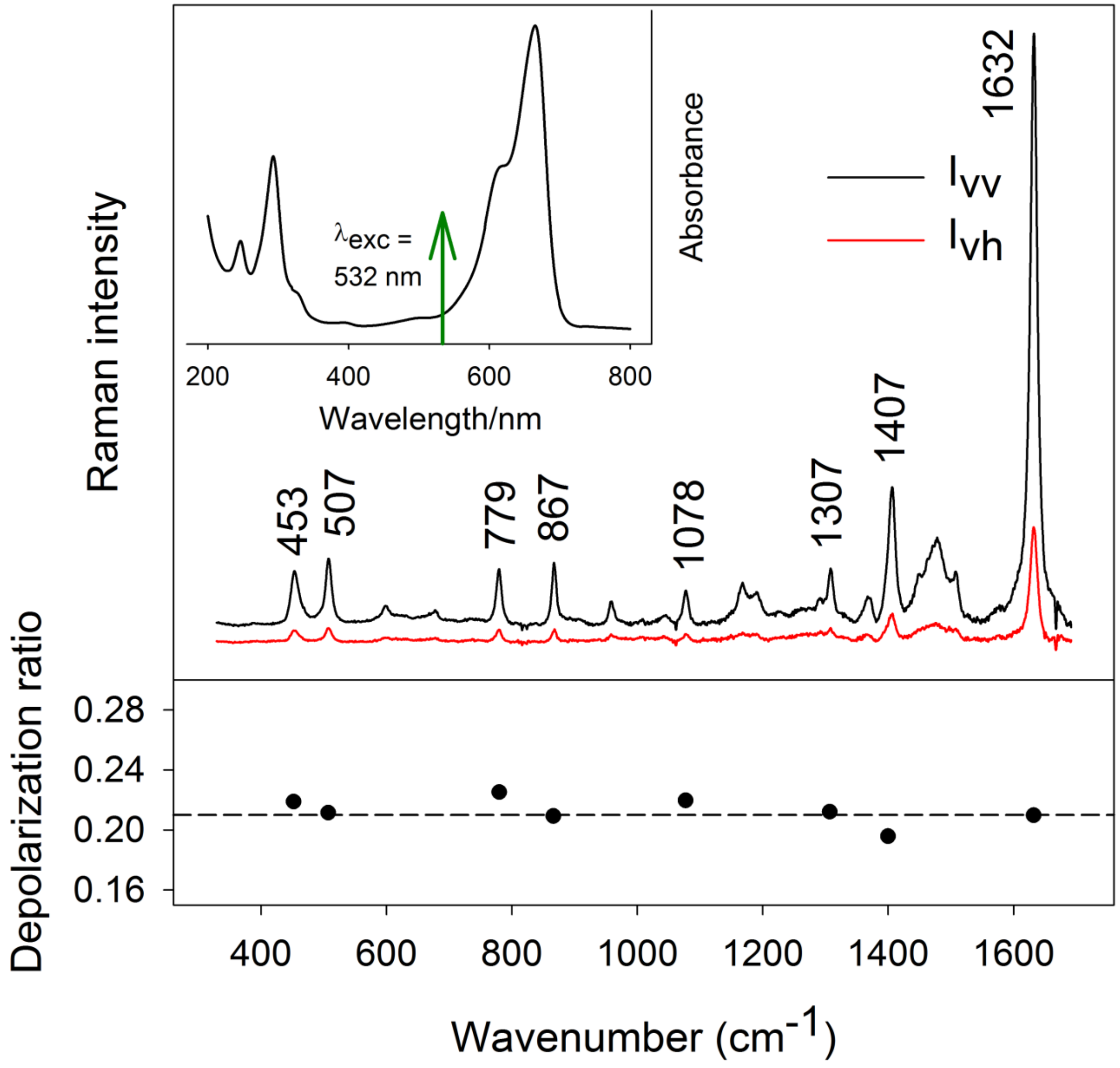

Figure S2. Pre-resonance Raman spectra of MB measured under non-SERS conditions in the vv (parallel-polarized) and vh (cross-polarized) arrangement. Presented spectra are after fluorescence background subtraction and offset for clarity. The MB concentration was 10^{-5} M, Inset: Absorption spectra of MB with the exciting wavelength marked with a green arrow. The depolarization ratios of the most intense A-type bands are presented below and take the value $\rho_M = 0.21 \pm 0.01$ as indicated by the dashed line.

3. Details of Raman Experiments

Since the obtained polarization and angular characteristics depend on a wide range of factors, we first investigated the effect of the geometrical layout, *i.e.* effectiveness of collection of the scattered radiation and the effect of the laser spot size with a given angle θ. Fig. S3 demonstrates the difference between experimentally measured Raman intensities of a Si wafer and corresponding theoretical values predicted by the surface selection rules, both with the changing inclination angle θ measured in the vv arrangement. For this purpose, optical pseudo-refractive index of a Si wafer $\tilde{n} = n + ik$ was obtained using spectral ellipsometry as $n = 4.11$, $k = 0.35$ (for $\lambda = 532$ nm) and $n' = 4.05$, $k' = 0.33$ for $\lambda' = 547$ nm (corresponding to the wavelength of the scattered radiation of the 520-cm^{-1} silicon mode). The vv arrangement was chosen due to the fact that corresponding intensities depend only on one term of the Raman tensor. Comparison between experimental and theoretical values implies that their ratio can be well fitted by an expected $\cos\theta$ function[4] and suggests that very similar effect (variation in the laser spot size with the angle θ) will play a dominant role also in the case of AgOADs. To eliminate this effect, SERS depolarization ratios were further analyzed instead of intensities (see the main text).

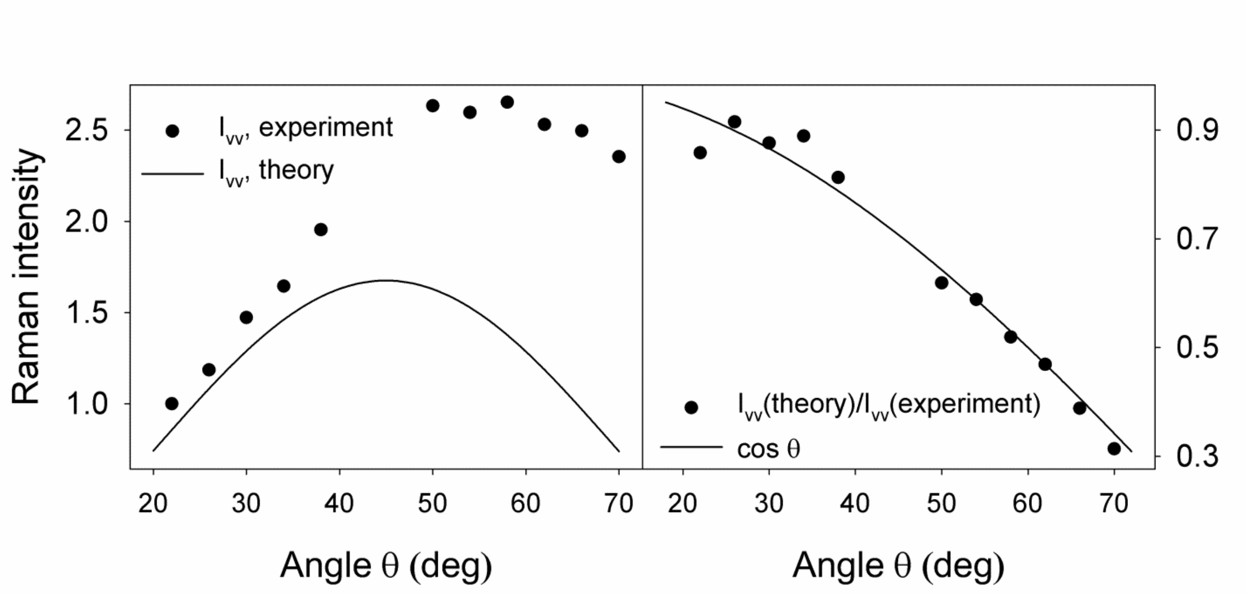

Figure S3. Left: Comparison between Raman intensities of a Si wafer (black points) and (normalised) theoretical values predicted by the surface selection rules (line) with the varying inclination angle θ as measured in the vv arrangement. Right: The ratios of theoretical/experimental values from the left graph and their fit by a $\cos\theta$ function.

4. Modelling of the Plasmonic Anisotropy

At the first stage of the analysis of the optical response of the AgOADs, a simple model based on "competition" between the enhancement provided by longitudinal plasmon modes and by transverse plasmon modes was employed. This model makes use of the fact that the plasmonic response of light polarized parallel/perpendicular to the nanorod axis should be different. Since the projection of incident/scattered polarization to the direction of growing nanorods is given by the scalar product between these two vectors, the intensities obtained in respective cases (varying angles θ and φ) are expected to be proportional to the right side of eq 8 (for simplicity, we will now suppose the factors $\mathrm{a}_{||}$ and $\mathrm{a}_{\perp}$ the same both for the exciting wavelength and the Raman-shifted wavelength). Since we analyse the depolarization ratios instead of intensities, the final formulas are expected to depend only on the ratio $r = \mathrm{a}_{\perp}/\mathrm{a}_{||}$. Fig. S4 clearly demonstrates that no value of r consistent for all experimental configurations can be found. The reasons are as follows: (i) any possible value of r (irrespective whether $r < 1$ or $r > 1$) is able to consistently copy the shape of all 3 experimentally measured depolarization ratios, (ii) for $r > 1$, the depolarization ratios for $\varphi = 0°$ and $\varphi = 180°$ lie below the depolarization ratios corresponding to $\varphi = 90°$ and $\varphi = 270°$, while from experiment the depolarization ratio curves for $\varphi = 0°$ and $\varphi = 180°$ are above those for $\varphi = 90°$ and $\varphi = 270°$, (iii) even for $r < 1$, the two depolarization ratio curves especially for $\varphi = 0°$ and $\varphi = 180°$ are (supposedly) distinct (the distinction being the more pronounced the lower is r), although in experiment they are almost identical, (iv) the increasing/decreasing trend in the depolarization ratio curves for ρ_1/ρ_2 is not compatible with the model supposing plasmonic anisotropy of our structures. Nor is the U-shaped trend in the case of experimentally measured ρ_3 since the model supposing plasmonic anisotropy predicts very little dependence on the angle θ in this case. Most importantly, the model based on plasmonic anisotropy predicts the existence of three different trends for the experimentally measured depolarization ratio curves (since the angles $\varphi = 90°$ and $\varphi = 270°$ provide the same response in the plasmonic anisotropy model) while in reality only two distinct trends were measured (see Fig. 5). In conclusion, we suggest that although our nanostructures are morphologically anisotropic, the plasmonic properties around the wavelength used (532 nm) are rather isotropic and therefore they are not responsible for the anisotropic behaviour we observed in the SERS experiments.

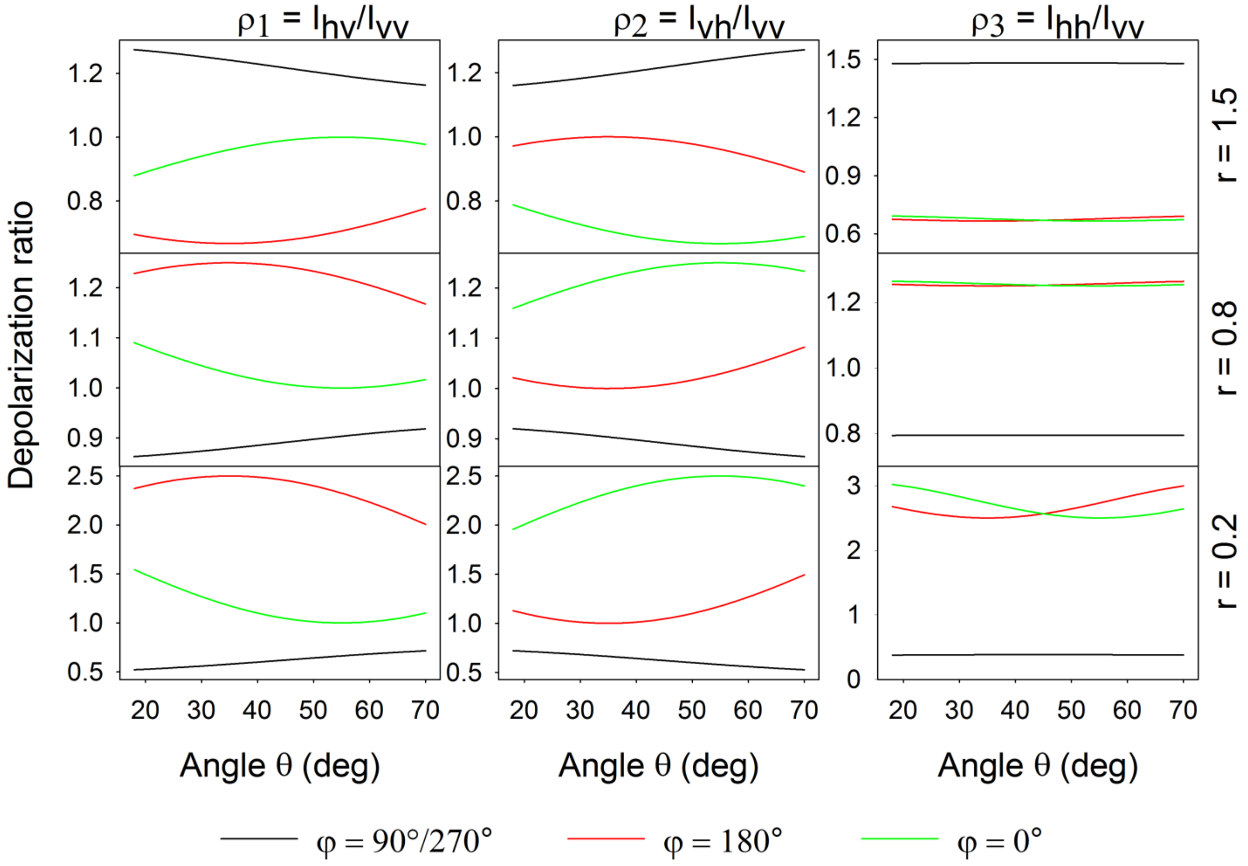

Figure S4. Theoretical shapes of the depolarization ratio curves in the case of plasmonic anisotropy described by the r value. Three values of r, three depolarization ratios and three angles φ (since the angles $\varphi = 90°$ and $\varphi = 270°$ provide the same response in the plasmonic anisotropy model) are presented. Since obviously no value of r is able to fit the experimentally measured depolarization curves, the plasmonic anisotropy around the wavelength 532 nm is not the main reason for anisotropic behaviour observed in the SERS experiments.

5. Spectral Ellipsometry Measurements

Spectral ellipsometry measures changes in light polarization after reflecting from a material structure. To describe reflection from the surface, it is traditional to resolve the electric field into two orthogonal components: parallel (p) and perpendicular (s) with respect to the plane of incidence. Both the amplitude and the phase of both field components undergo a change upon reflection as determined by the Fresnel reflection coefficients

$$\tilde{r}_s = \frac{\cos\theta - \sqrt{\tilde{n}^2 - \sin^2\theta}}{\cos\theta + \sqrt{\tilde{n}^2 - \sin^2\theta}}, \qquad \text{(S3)}$$

$$\tilde{r_p} = \frac{\tilde{n}^2 \cos\theta - \sqrt{\tilde{n}^2 - \sin^2\theta}}{\tilde{n}^2 \cos\theta + \sqrt{\tilde{n}^2 - \sin^2\theta}}, \tag{S4}$$

where θ denotes the angle of incidence. Complex quantities are marked with a tilde. A commonly measured ellipsometric quantity describing changes in polarization upon reflection is the complex reflectance ratio, defined as

$$\tilde{\rho} = \frac{\tilde{r_s}}{\tilde{r_p}}. \tag{S5}$$

The inverse transformation is

$$\tilde{n}^2 = \sin^2\theta \left[1 + \tan^2\theta \left(\frac{1-\tilde{\rho}}{1+\tilde{\rho}}\right)^2\right], \tag{S6}$$

which allows to compute the pseudo-refractive index of the structure, knowing the complex reflectance ratio $\tilde{\rho}$. Both $\tilde{\rho}$ and $\tilde{n}$ depend inherently on light frequency.

For semi-infinite, homogeneous and isotropic samples, the pseudo-refractive index is independent of the incident angle, which means it is sufficient to measure $\tilde{\rho}$ only for one angle θ for unique description of the reflective properties of the material. Then, the pseudo-refractive index becomes identical with the refractive index of the material. However, the above-mentioned criteria are hardly ever met, often due to surface roughness, native oxide layers (the case of a Si wafer) or presence of other films and overlays. Therefore, the pseudo-refractive index of silicon may be different from its refractive index. Moreover, in the case of nanostructured materials, the assumption that the pseudo-refractive index is independent of the incident angle does not have to hold anymore, which is due to the presence of subwavelength structures (see Figs. 6 and S5).

In a more general case such as anisotropic, but still non-depolarizing materials, it is convenient to make use of the Jones formalism and express the components of the reflected light in the matrix form

$$\begin{pmatrix} E_r^p \\ E_r^s \end{pmatrix} = \begin{pmatrix} r_{pp} & r_{ps} \\ r_{sp} & r_{ss} \end{pmatrix} \begin{pmatrix} E_i^p \\ E_i^s \end{pmatrix}, \tag{S7}$$

where the subscript i stands for the incident and r for the reflected radiation. Generalized ellipsometry measures the ratios of the Jones matrix elements, *i.e.*

$$\frac{r_{pp}}{r_{ss}}, \frac{r_{ps}}{r_{pp}}, \frac{r_{sp}}{r_{ss}}, \tag{S8}$$

the two latter of them being identically zero in the case of homogeneous and isotropic samples.

Finally, samples which exhibit depolarization are not compatible with standard or generalized ellipsometry measurements and thus should be treated by the Mueller-matrix formalism. This formalism employs Stokes vectors and 4×4 matrices to account for depolarization, *i.e.* loss of

coherence of the phase and amplitude of the electric field.[5] The conversion of the polarized light into partially polarized or unpolarized happens due to surface roughness, film thickness inhomogeneity or presence of plasmonic resonances. Occurring depolarization may be surmised from the comparison between Mueller-matrix and Jones-matrix elements or between reflection intensities and corresponding intensities calculated using Jones-matrix elements.

Generalized ellipsometry measurements of AgOADs showed that the ratios $\frac{r_{ps}}{r_{pp}}, \frac{r_{sp}}{r_{ss}}$ are < 0.01 (not shown here) and thus the non-diagonal terms of the Jones matrix were neglected in our calculations. We expect that this is due to rather densely packed nanocolumns and low porosity of the AgOADs. Measurements in the reflection mode further revealed that our structures may be treated (around the wavelength of 532 nm) as non-depolarizing and thus analyzed by the standard Jones formalism. Fig. S6 demonstrates the difference between total reflectivity as measured in the reflection mode and reflectivity computed via optical pseudo-parameters retrieved from standard ellipsometry measurements using the Fresnel equations. As expected, the values obtained by direct reflection measurements are for most angles slightly higher than that computed, which do not take depolarization into account. The average difference between these two methods is around 5%. The slight discrepancy at small angles θ and $\varphi = 90°/270°$ may be caused by the low values of the imaginary part of the refractive index which implies high penetration depth of the incident angle through the nanostructures, which means that the effective reflection does not occur only at the interface Ag/air, but also at the interface Ag/Si. Upon reflection, however, the part of the light reflected from the Si interface is absorbed in the material structure and that is why the approach based on the effective medium approximation somewhat overestimates the total reflectivity.

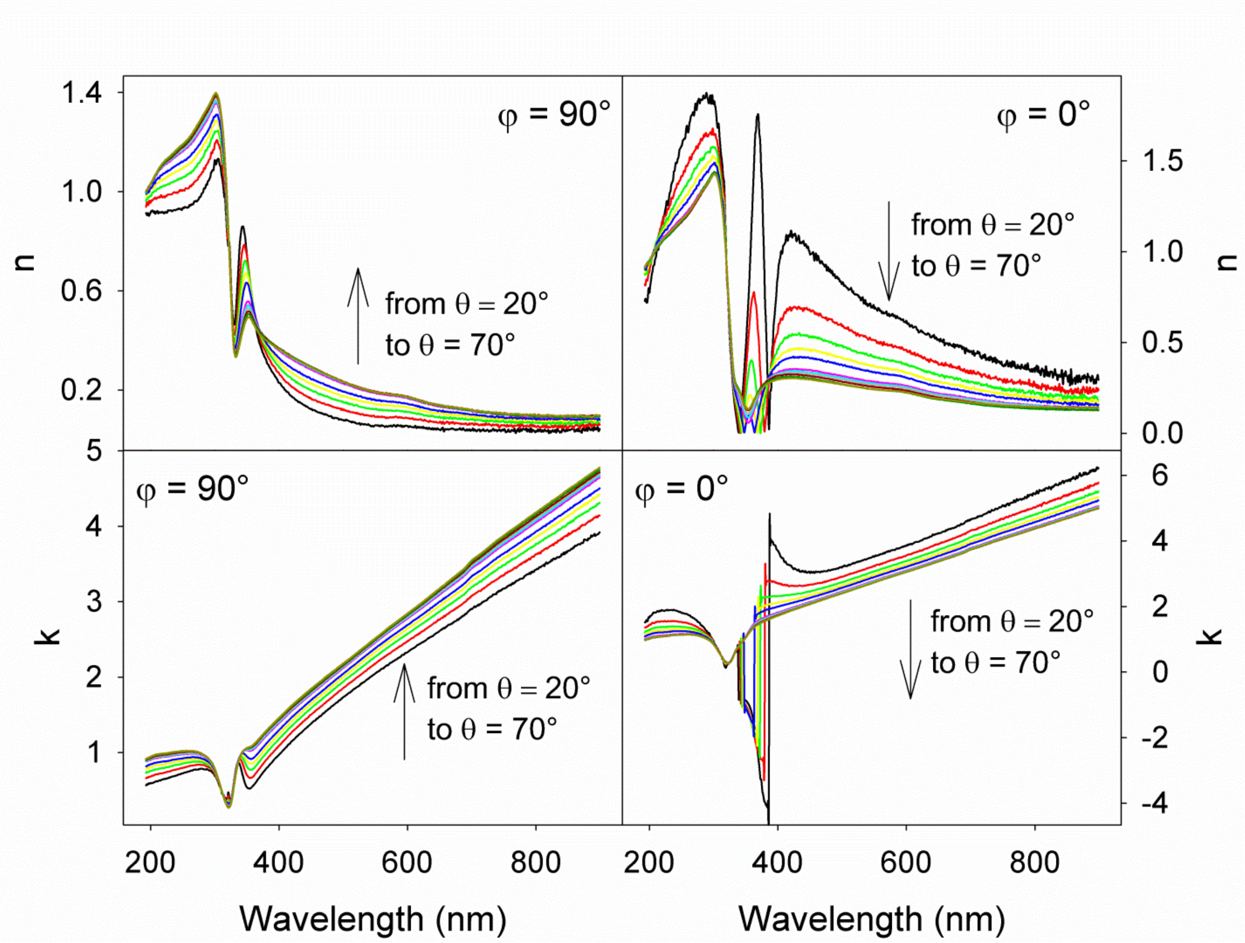

Figure S5. Wavelength dependence of the real and imaginary part of the pseudo-refractive index of AgOADs for different angles of incidence θ. Left: $\varphi = 90°$ (virtually identical to $\varphi = 270°$), right: $\varphi = 0°$ (virtually identical to $\varphi = 180°$). Variation with the angle of incidence θ around $\lambda = 500$ nm is indicated by arrows.

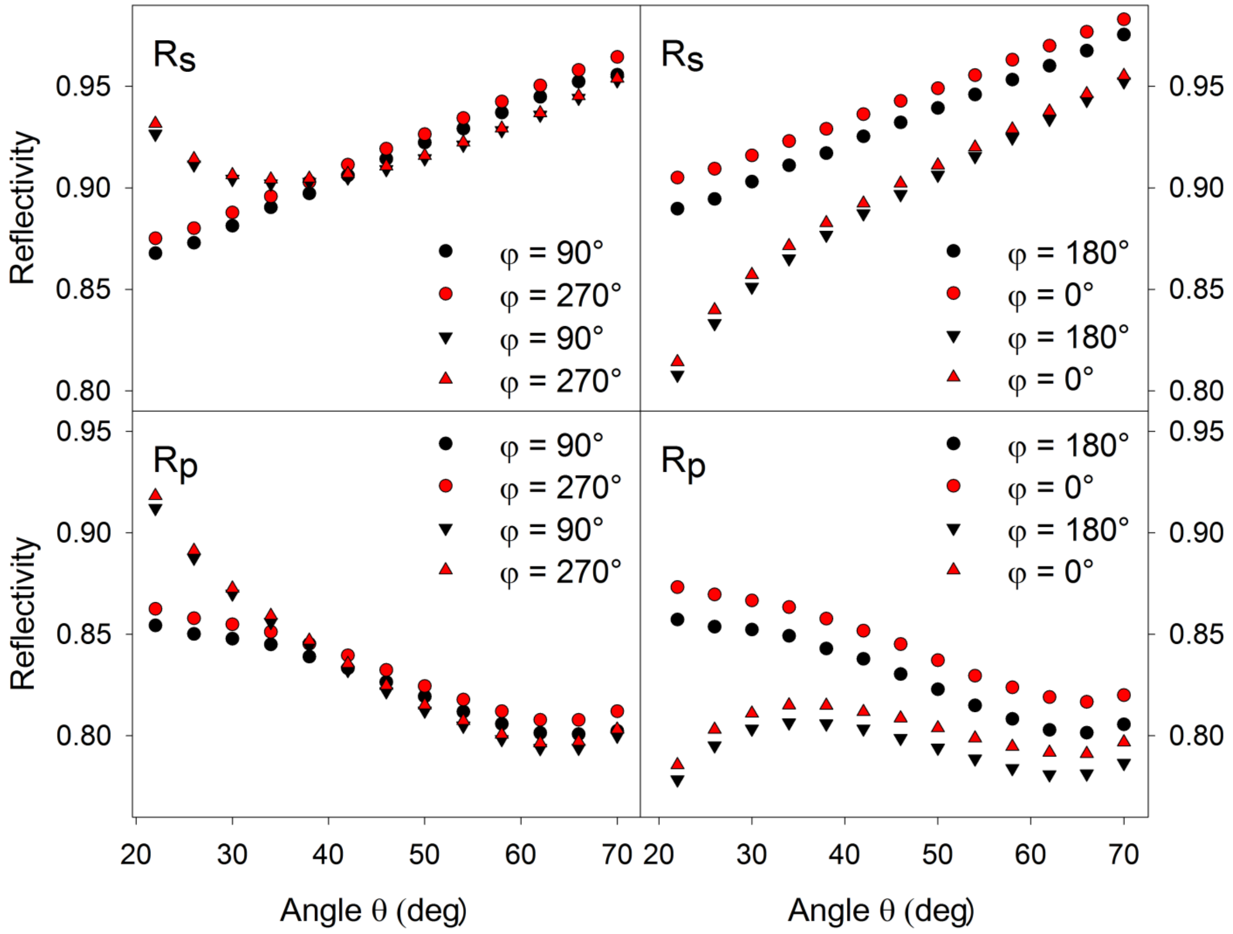

Figure S6. Reflectivity of the AgOADs as measured using reflection mode (circles) and as computed using pseudo-refractive indices measured in the standard ellipsometry mode (triangles). The values R_s are in the two graphs above, the values R_p are below; $\lambda = 532$ nm.

6. Surface Selection Rules

Let us consider the situation when a molecule adsorbed on a surface (determined by the $x'y'$ plane and z' specifying the substrate normal) is irradiated by a light beam at an incident angle θ with the wavevector perpendicular to the x' axis. Let the scattered radiation be collected at an angle θ', again with the wavevector perpendicular to x'. The molecule may be thought of as being irradiated by two beams: The direct (incident) and the reflected one, which superimpose coherently, and a similar process applies in the case of the scattered radiation. In the following, we will denote $\vec{e}$ the unit electric field vector, the subscripts i and r stand for the incident and reflected radiation respectively, and primed quantities pertain to the scattered radiation. This situation is depicted in Fig. S7.

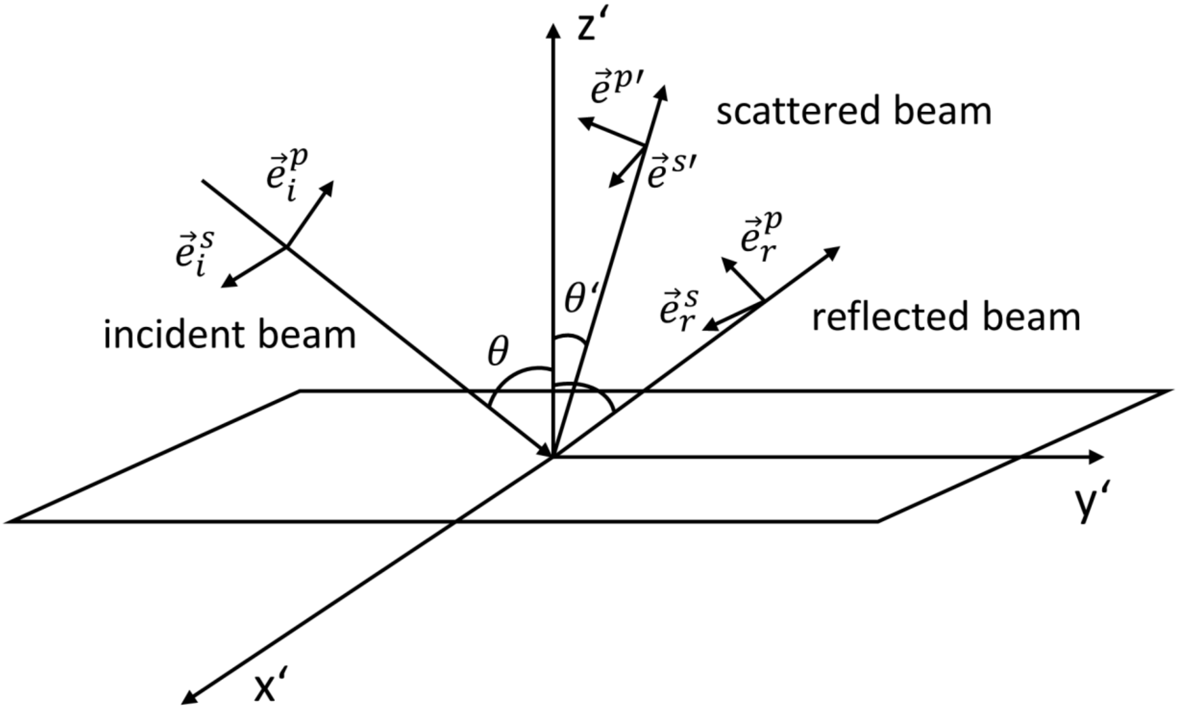

Figure S7. Scheme of the geometrical layout and definition of the coordinates. All symbols have usual meanings as described in the text. The components of the scattered field experiencing a reflection from the surface ($\vec{e}_r^{s'}$ and $\vec{e}_r^{p'}$) are not included in the scheme for simplicity.

First, we will address the description of the incident beam. In the case of s-polarization, we have

$$\vec{e}_i^s = (1, 0, 0),\ \vec{e}_r^s = (r_s, 0, 0), \tag{S9}$$

and thus the total field felt by the molecule is

$$\vec{e}^s = (1 + r_s, 0, 0). \tag{S10}$$

In the case of p-polarization, we have

$$\vec{e}_i^p = (0, \cos\theta, \sin\theta),\ \vec{e}_r^p = \left(0, -r_p\cos\theta, r_p\sin\theta\right), \tag{S11}$$

and the total field felt by the molecule is

$$\vec{e}^p = \left(0, \left(1 - r_p\right)\cos\theta, (1 + r_p)\sin\theta\right). \tag{S12}$$

Very similar principle applies in the case of the scattered radiation. Supposing an analyser allows only the detection of the s-polarized light, we have

$$\vec{e}^{s'} = (1, 0, 0),\ \vec{e}_r^{s'} = (r_s', 0, 0), \tag{S13}$$

and the total detected field is

$$\vec{e}^{s'} = (1 + r_s^{'}, 0, 0). \tag{S14}$$

Finally, for the p-polarized light entering the detector, we have

$$\vec{e}^{p'} = (0, -\cos\theta', \sin\theta'),\ \vec{e}_r^{p'} = \left(0, r_p^{'}\cos\theta', r_p^{'}\sin\theta'\right), \tag{S15}$$

and the total detected field is

$$\vec{e}^{p'} = \left(0, \left(r_p^{'} - 1\right)\cos\theta', (1 + r_p^{'})\sin\theta'\right). \tag{S16}$$

Raman scattering intensity is proportional to

$$(e_j^{'}\alpha_{jk}^{'}e_k)^2, \tag{S17}$$

which means that, for example, the Raman scattering intensity of the p-polarized light excited by the s-polarized light will be proportional to

$$\left|\left(0, \left(r_p^{'} - 1\right)\cos\theta', (1 + r_p^{'})\sin\theta'\right) \cdot \begin{pmatrix} \alpha_{xx}^{'} & \alpha_{xy}^{'} & \alpha_{xz}^{'} \\ \alpha_{yx}^{'} & \alpha_{yy}^{'} & \alpha_{yz}^{'} \\ \alpha_{zx}^{'} & \alpha_{zy}^{'} & \alpha_{zz}^{'} \end{pmatrix} \cdot \begin{pmatrix} 1 + r_s \\ 0 \\ 0 \end{pmatrix}\right|^2, \tag{S18}$$

which produces the result given in eq 6. We abbreviate this intensity as I_{vh} with the first subscript standing for the exciting light (*i.e.* “vertical“ with respect to the scattering plane) and the second subscript standing for the scattered light (*i.e.* “horizontal“ with respect to the scattering plane). Intensity profiles for the three remaining polarization combinations may be derived in a very similar fashion and are summarized in the main text.

7. Raman Tensor Invariants and the Depolarization Ratio

Tensor elements are transformed upon rotation of axes. Denoting $\alpha_{ij}^{'}$ Raman tensor elements in a certain (primed) system of Cartesian coordinates and $\alpha_{ij}^{''}$ Raman tensor elements in another (double primed) system of Cartesian coordinates, the 9 components of the 3×3 matrix will be transformed according to the formula[6]

$$\alpha_{kl}^{''} = R_{ki}R_{lj}\alpha_{ij}^{'}. \tag{S19}$$

More explicitly, assuming that R is the rotational matrix about the z' axis by 90°, *i.e.* taking the form

$$R = \begin{pmatrix} \cos\omega & \sin\omega & 0 \\ -\sin\omega & \cos\omega & 0 \\ 0 & 0 & 1 \end{pmatrix}_{\omega=90°} = \begin{pmatrix} 0 & 1 & 0 \\ -1 & 0 & 0 \\ 0 & 0 & 1 \end{pmatrix}, \qquad \text{(S20)}$$

then, the transformation relation (S19) reads

$$\begin{pmatrix} \alpha''_{xx} & \alpha''_{xy} & \alpha''_{xz} \\ \alpha''_{yx} & \alpha''_{yy} & \alpha''_{yz} \\ \alpha''_{zx} & \alpha''_{zy} & \alpha''_{zz} \end{pmatrix} = \begin{pmatrix} 0 & 1 & 0 \\ -1 & 0 & 0 \\ 0 & 0 & 1 \end{pmatrix} \cdot \begin{pmatrix} \alpha'_{xx} & \alpha'_{xy} & \alpha'_{xz} \\ \alpha'_{yx} & \alpha'_{yy} & \alpha'_{yz} \\ \alpha'_{zx} & \alpha'_{zy} & \alpha'_{zz} \end{pmatrix} \cdot \begin{pmatrix} 0 & -1 & 0 \\ 1 & 0 & 0 \\ 0 & 0 & 1 \end{pmatrix} =$$

$$\begin{pmatrix} \alpha'_{yy} & -\alpha'_{yx} & \alpha'_{yz} \\ -\alpha'_{xy} & \alpha'_{xx} & -\alpha'_{xz} \\ \alpha'_{zy} & -\alpha'_{zx} & \alpha'_{zz} \end{pmatrix}. \qquad \text{(S21)}$$

Leaving aside the unimportant signs in the final matrix product in eq S21, we see that the Raman tensor component α'_{xx} for $\varphi = 90°/270°$ becomes identical to α'_{yy} for $\varphi = 0°/180°$ and vice versa, the Raman tensor component α'_{xz} for $\varphi = 90°/270°$ becomes α'_{yz} for $\varphi = 0°/180°$ (and vice versa) etc.

In the case of resonance Raman scattering, a total of 3 independent combinations of Raman tensor elements can be found that remain invariant after rotation of the system of coordinates. These are usually referred to as:[6]

The square of the mean polarizability a:

$$a^2 = \frac{(\alpha_{xx} + \alpha_{yy} + \alpha_{zz})^2}{9}, \qquad \text{(S22)}$$

the anisotropy γ:

$$\gamma^2 = \frac{(\alpha_{xx} - \alpha_{yy})^2 + (\alpha_{yy} - \alpha_{zz})^2 + (\alpha_{xx} - \alpha_{zz})^2}{2} + 3\frac{(\alpha_{xy} + \alpha_{yx})^2 + (\alpha_{yz} + \alpha_{zy})^2 + (\alpha_{zx} + \alpha_{xz})^2}{4}, \qquad \text{(S23)}$$

and the antisymmetric anisotropy δ:

$$\delta^2 = 3\frac{(\alpha_{xy} - \alpha_{yx})^2 + (\alpha_{yz} - \alpha_{zy})^2 + (\alpha_{zx} - \alpha_{xz})^2}{4}. \qquad \text{(S24)}$$

The relative proportion between these 3 invariants (especially between a^2 and γ^2 since δ^2 tends to zero in the case of non-resonance Raman scattering) helps to surmise the symmetry of the vibration involved. In a typical experiment, this information is accessible with the use of polarized light. For the sake of simplicity, we limit our attention here to the 90°-scattering geometry, although some of the following results may be easily generalized to other geometries too. As already mentioned in the section 2, in the case of randomly oriented molecules, the ratio of cross-polarized (in principle, any of I_{hv}, I_{vh} or I_{hh} polarization arrangements) to parallel-polarized (I_{vv} arrangement) Raman intensities defines the Raman depolarization ratio ρ.

Equivalently, it involves the ratio of the orientation-averaged non-diagonal Raman tensor components to the diagonal ones, which may be expressed using the three above-mentioned invariants as

$$\rho = \frac{\langle \alpha_{ij}^2 \rangle}{\langle \alpha_{ii}^2 \rangle} = \frac{3\gamma^2 + 5\delta^2}{4\gamma^2 + 45a^2}. \quad \text{(S25)}$$

We expect that the difference between the depolarization ratio of MB as measured in a water solution ($\rho_M = 0.21 \pm 0.01$) and that computed for MB adsorbed on AgOADs using eq S25 and values from table 1 ($\rho = 0.29 \pm 0.01$) is attributable mainly to the presence of hot-spot sites. Briefly, in the following, we will assume random orientation of dimers (which are generally agreed to be the principal sources of hot-spot sites) between which a molecule may be embedded. Then, the local field scales as ~ $\cos\alpha$ where α is the angle between polarization vector and the dimer axis. Introducing standard spherical coordinates $(x,\ y,\ z) = (\sin u \cos v\,, \sin u \sin v, \cos u)$, we obtain

$$\rho_{SERS} = \frac{I_{vh}}{I_{vv}} = \frac{I\left(e_x^{exc}, e_z^{det}\right)}{I\left(e_x^{exc}, e_x^{det}\right)} = \frac{\iint (\sin u \cos u \cos v)^2 \mathrm{d}\Omega}{\iint (\sin u \cos v)^4 \mathrm{d}\Omega}, \quad \text{(S26)}$$

where $\mathrm{d}\Omega = \sin u \, \mathrm{d}u\mathrm{d}v,\ u \epsilon (0, \pi)$ and $v \epsilon (0, 2\pi)$. Simple integration yields the value of $\rho_{SERS} = 1/3$ as verified experimentally many times.[7,8] As expected, the depolarization ratio as computed using values in table 1 is bound between these two extremes ($\rho_M < \rho < \rho_{SERS}$). Another reason for the difference between ρ and ρ_M may be formation of dimers/trimers at the surface, which could feature a different depolarization ratio with respect to the monomers that are analyzed in liquid (or possibly a combination of the two factors mentioned above).

References

1. Malinowski, E.R. *Factor Analysis in Chemistry* (J. Wiley & Sons. 2002).
2. Roy, S. D., Ghosh, M. & Chowdhury, J. Adsorptive parameters and influence of hot geometries on the SER(R) S spectra of methylene blue molecules adsorbed on gold nanocolloidal particles. *J. Raman Spectrosc.* **46**, 451-461 (2015).
3. Heger, D., Jirkovský, J. & Klán, P. Aggregation of Methylene Blue in Frozen Aqueous Solutions Studied by Absorption Spectroscopy. *J. Phys. Chem. A* **109**, 6702-6709 (2005).
4. Le Ru, E. C. *et al*. Experimental demonstration of surface selection rules for SERS on flat metallic surfaces. *Chem. Commun.* **47**, 3903-3905 (2011).
5. Jellison, G. E. Spectroscopic ellipsometry data analysis: measured versus calculated quantities. *Thin Solid Films* **313**, 33-39 (1998).
6. Long, D. A. *The Raman Effect: A Unified Treatment of the Theory of Raman Scattering by Molecules* (John Wiley & Sons Ltd, 2002).

7. Le Ru, E. C., Meyer, M., Blackie, E. & Etchegoin, P. G. Advanced aspects of electromagnetic SERS enhancement factors at a hot spot. *J. Raman Spectrosc.* **39**, 1127-1134 (2008).
8. Fazio, B. *et al*. Re-radiation Enhancement in Polarized Surface-Enhanced Resonant Raman Scattering of Randomly Oriented Molecules on Self-Organized Gold Nanowires. *ACS Nano* **5**, 5945-5956 (2011).
